# One-Pot Synthesis of Hierarchical Flower-Like Pd-Cu Alloy Support on Graphene Towards Ethanol Oxidation

**DOI:** 10.1186/s11671-017-2290-7

**Published:** 2017-09-02

**Authors:** Jingyi Zhang, Anni Feng, Jie Bai, Zhibing Tan, Wenyao Shao, Yang Yang, Wenjing Hong, Zongyuan Xiao

**Affiliations:** State Key Laboratory of Physical Chemistry of Solid Surfaces, Collaborative Innovation Center of Chemistry for Energy Materials, Department of Chemical and Biochemical Engineering, College of Chemistry and Chemical Engineering, Xiamen University, Xiamen, 361005 China

**Keywords:** Pd nanocatalyst, Flower-like, Ethanol oxidation, Direct ethanol fuel cells

## Abstract

The synergetic effect of alloy and morphology of nanocatalysts play critical roles towards ethanol electrooxidation. In this work, we developed a novel electrocatalyst fabricated by one-pot synthesis of hierarchical flower-like palladium (Pd)-copper (Cu) alloy nanocatalysts supported on reduced graphene oxide (Pd-Cu_(F)_/RGO) for direct ethanol fuel cells. The structures of the catalysts were characterized by using scanning electron microscopy (SEM), transmission electron microscope (TEM), X-ray diffraction (XRD), and X-ray photoelectron spectrometer (XPS). The as-synthesized Pd-Cu_(F)_/RGO nanocatalyst was found to exhibit higher electrocatalytic performances towards ethanol electrooxidation reaction in alkaline medium in contrast with RGO-supported Pd nanocatalyst and commercial Pd black catalyst in alkaline electrolyte, which could be attributed to the formation of alloy and the morphology of nanoparticles. The high performance of nanocatalyst reveals the great potential of the structure design of the supporting materials for the future fabrication of nanocatalysts.

## Background

Direct ethanol fuel cells (DEFCs) are considered as economic and environment-friendly renewable energy sources because of the low operating temperature, renewability, low toxicity, and high energy density [1, 2]. Long-term activity has remained as a tremendous challenge for future application of DEFCs, while the poisoning becomes the bottleneck for further improvement. Among all metal nanocatalysts, Pd attracts more attention not only because of its lower cost but also its little CO poisoning effects for electrochemical oxidation of ethanol [3, 4]. In addition, it is reported that morphology and structures of supporting materials or nanoparticles might significantly influence their electrochemistry properties [5, 6], and the following have been studied: micro/nanoleaves [7], nanoflowers [6], nanowires [8], hierarchical hollow microsphere [9], and flower/grass-like structures [10]. The hierarchical flower-like copper is recently reported by changing the morphology of copper to acquire a large surface area [5, 11, 12]. It is also found that copper not only reduce the cost of electrocatalyst but also can be more preferable for the adsorption of hydroxyls, which further increases the rate of alcohol oxidation [4, 13]. Moreover, the electronic properties would be changed due to the d-band center shifts during the formation of Pd-Cu alloy, and the synergistic effect of the composition further increase the electrocatalytic activity towards ethanol [14, 15].

Besides metallic materials, the ideal supporting materials of electrocatalysts are expected to have high surface area and good electrical conductivity [16, 17], and recently, series of graphene and its complex materials are developed as supported materials for the nanocatalysts towards ethanol oxidation. It was reported that metals could be well dispersed on the graphene due to the large numbers of functional groups on the graphene layers that exhibited high catalytic activity for electrooxidation of alcohol [15, 16, 18]. Therefore, it would be promising to develop a flower-like Pd-Cu alloy nanocatalysts supported on reduced graphene oxide towards electrochemical oxidation of ethanol.

Herein, we developed a facile one-pot hydrothermal approach to prepare flower-like Pd-Cu alloy nanoparticles supported on reduced graphene oxide (RGO). The addition of ammonia solution not only influence the formation of Pd-Cu alloy but also lead to the hierarchical flower-like structure, attaching to the RGO surface, synergistically increasing the surface area of electrocatalysts and acquiring more available active sites [19]. The Pd-Cu_(F)_/RGO nanocatalyst was characterized by scanning electron microscopy (SEM), transmission electron microscope (TEM), X-ray diffraction (XRD), X-ray photoelectron spectrometer (XPS), inductively coupled plasma optical emission spectroscopy (ICP-OES), and thermogravimetric analysis (TGA). The electrochemical studies in alkaline medium show that the Pd-Cu_(F)_/RGO nanocatalyst provides higher activity and significantly better long-term activity towards ethanol electrooxidation than RGO-supported Pd nanocatalyst and commercial Pd black.

## Methods

### Reagent and Chemicals

Copper(II) nitrate trihydrate (Cu(NO_3_)_2_·3H_2_O), palladium chloride (PdCl_2_), ethylene glycol (EG), ethanol, graphite powder (S.P.), sulfuric acid (98 wt% H_2_SO_4_), potassium permanganate (KMnO_4_), and potassium borohydride (95 wt% KBH_4_) were purchased from Sinopharm Chemical Reagent Co., Ltd. Hydrogen peroxide (30 wt% H_2_O_2_) and ammonia solution were provided by Guangdong Guanghua Sci Tech Co., Ltd. Sodium hydroxide (NaOH) was offered by Aladdin Industrial Inc. Polyvinylpyrrolidone (PVP, MW = 30,000, A.R.) was purchased from Sinopharm Chemical Reagent Co., Ltd. (Shanghai, China). Ten percent of Pd black was provided by HESEN Electric Co., Ltd. (Shanghai, China). Five weight percent of Nafion solution was obtained from Sigma Aldrich. All chemicals were used without any further purification.

### Preparation of the Pd-Cu_(F)_/RGO

#### Preparation of Graphene Oxide (GO)

GO was prepared from graphite powder according to a modified Hummers method [20].

#### Preparation of Pd-Cu_(F)_/RGO

Firstly, a mixed solution of 40 mL EG and 40 mL ethanol was prepared, and 160 mg of PVP was put into the solution under sonication for 30 min, then adding 0.01 mol L^− 1^ PdCl_2_ and 0.02 mol L^− 1^ Cu(NO_3_)_2_·3H_2_O into the mixed solution under stirring, following by adding a certain volume of ammonia solution to adjust pH = 10.0. Next, 30 mg of as-prepared GO was dispersed in the mixed solution of 5 mL EG and 5 mL ethanol under sonication condition to form GO suspension, then adding them to the solution mentioned above with sonication for another 60 min. After these steps, we transferred the mixture solution with 2 mL KBH_4_ (0.15 mg mL^− 1^) into a 50-mL Teflon-lined autoclave and maintained at 160 °C for 6 h. After cooling down to the room temperature, the product was centrifuged and washed for several times with ultrapure water and ethanol. At last, the product was dried at 40 °C in a vacuum overnight; the result was named Pd-Cu_(F)_/RGO.

We also prepared the spherical particle Pd and Cu supported on RGO nanocatalyst in the similar method mentioned above, while the difference is that ammonia solution was replaced by Na_2_CO_3_ solution. The catalysts obtained were marked as Pd-Cu_(P)_/RGO. And the RGO-supported Pd (Pd/RGO) or Cu (Cu_(F)_/RGO) nanocatalysts were also prepared under similar conditions without Cu(NO_3_)_2_·3H_2_O or PdCl_2_, respectively.

### Electrochemical Measurements

The electrochemical measurements for electrocatalytic activity and stability of catalysts were performed on an electrochemical work station CHI750D by using a three-electrode cell at room temperature. A platinum plate electrode was used as the counter electrode, while a saturated calomel electrode (SCE) was used as the reference electrode. The preparation of working electrode was the following steps: 2 mg of Pd-Cu_(F)_/RGO catalyst was added into 2 mL ultrapure water under sonication to form a suspension; then, 10 μL of the catalyst suspension was spread on the surface of a glassy carbon electrode (GCE, 5 mm in diameter), which has been carefully polished with alumina powers and cleaned with ultrapure water. Later, 5 μL Nafion solution (5 wt%) was dropped on the surface as a layer to cover the samples. For comparison, the Pd-Cu_(P)_/RGO, Pd/RGO, and commercial Pd black catalysts were also modified for the electrodes under the same conditions. In each experiment, high-purity nitrogen was used to saturate the electrolyte for 30 min to remove the oxygen.

## Results and Discussion

**Fig. 1:**
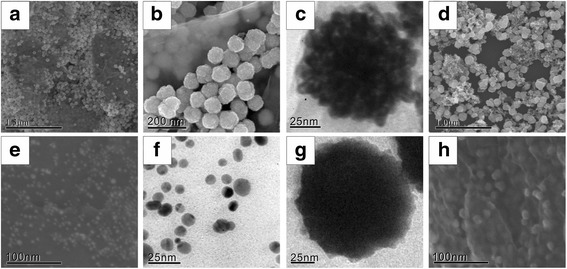
SEM (**a**, **b**) and TEM (**c**) images for Pd-Cu_(F)_/RGO. SEM (**d**) image for Pd-Cu_(F)_. SEM (**e**) and TEM (**f**) images for Pd-Cu_(P)_/RGO. TEM (**g**) image for Cu_(F)_/RGO. SEM (**h**) image for Pd/RGO

SEM and TEM were employed to investigate the size and morphology of Pd-Cu_(F)_/RGO, Pd-Cu_(P)_/RGO, Cu_(F)_/RGO, and Pd/RGO catalysts. As shown in Fig. 1a, the Pd-Cu alloy nanoparticles are on both sides of the graphene layer. It can be obviously seen from Fig. 1b, c that these Pd-Cu_(F)_/RGO nanocatalysts have a flower-like morphology which is different from particle Pd-Cu_(P)_/RGO nanoparticles shown in Fig. 1e, f. And the average particle sizes of these two catalysts were approximately 80 ± 5 nm and 10 ± 2 nm, respectively. The morphology of Pd-Cu_(F)_/RGO is much closer to Cu_(F)_/RGO nanoparticles shown in Fig. 1g rather than the spherical particle structure of Pd/RGO nanoparticles shown in Fig. 1h, and this compact hierarchical flower-like morphology is just as reported in previous studies, suggesting this structure is relevant to the impact of Cu and ammonia solution [21, 22]. The flower-like Pd-Cu nanoparticles without graphene were also fabricated shown in Fig. 1d to affirm the role of graphene in the electrocatalysts. It can be observed that the nanoparticles dispersed unevenly and some small nanoparticles aggregated together. Compare with the morphology of Pd-Cu_(F)_/RGO, it can be concluded that graphene is an ideal substrate for supporting and dispersing nanoparticles, which is consistent with previous reports [17, 21].

**Fig. 2:**
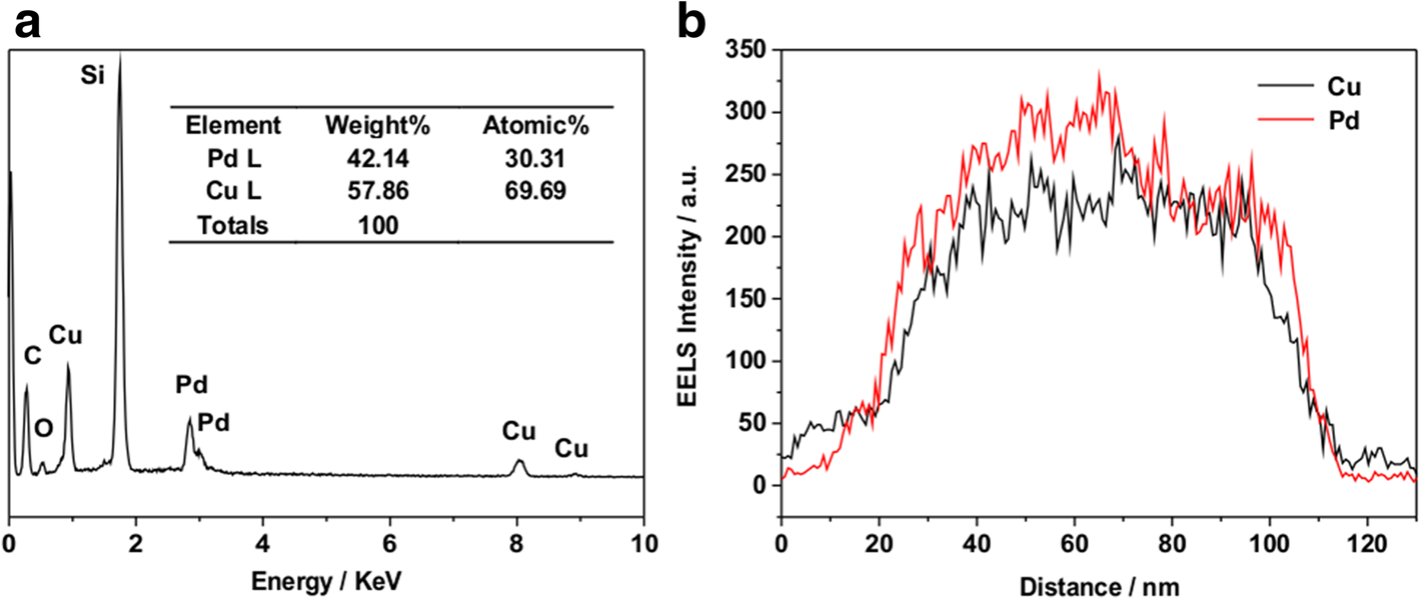
EDX spectra (**a**) and STEM-EDS profiles (**b**) of Pd-Cu_(F)_/RGO. The Si peak came from the necessary Si substrate

To investigate the element distribution of the Pd-Cu_(F)_/RGO, the energy dispersive X-ray spectroscopy (EDX) spectra was shown in Fig. 2a. The results indicated that the weight fractions of Pd and Cu of Pd-Cu_(F)_/RGO was approximately 1:1.4, which agreed with the feeding weight fractions of Pd and Cu which were 1:1.3. The actual weight fractions were further measured by inductively coupled plasma optical emission spectroscopy (ICP-OES), and the analysis result revealed that Pd-Cu_(F)_/RGO contains 15.8 wt% Pd and 21.4 wt% Cu, which was roughly consistent with EDX. The STEM-EDS scanning profiles (Fig. 2b) also indicated that Pd and Cu elements were loading on the catalysts homogeneously.

**Fig. 3:**
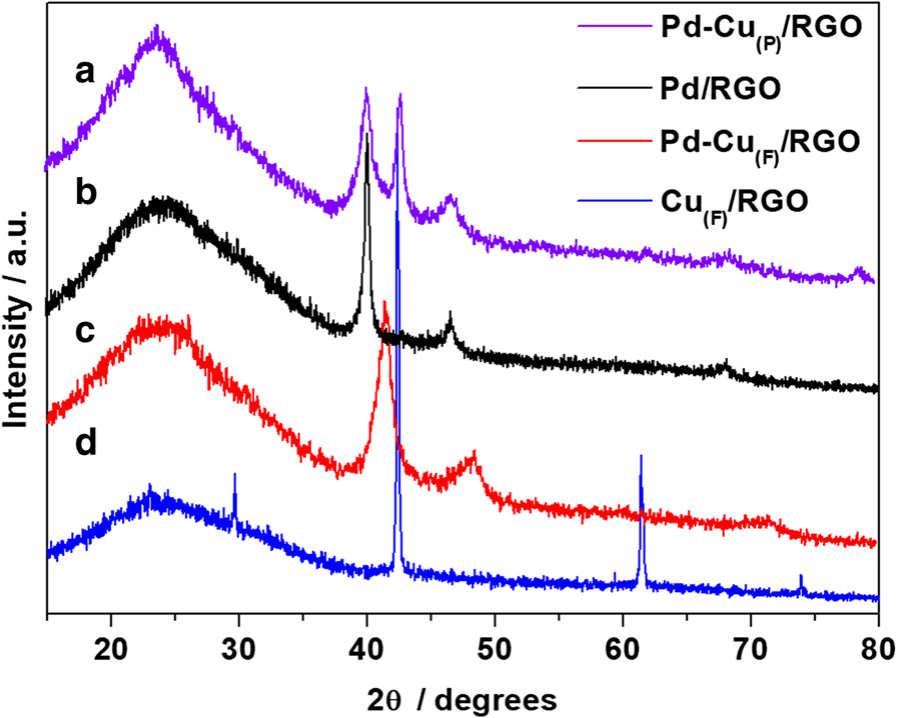
XRD patterns of the Pd-Cu_(P)_/RGO (curve a), Pd/RGO (curve b), Pd-Cu_(F)_/RGO (curve c), and Cu_(F)_/RGO (curve d)

The XRD patterns of the Pd-Cu_(F)_/RGO, Pd-Cu_(P)_/RGO, Pd/RGO, and Cu_(F)_/RGO catalysts were presented in Fig. 3. For the Pd-Cu_(P)_/RGO, three diffraction peaks were detected at 40.1°, 46.9°, and 68.6°, corresponding to the (111), (200), and (220) crystallite planes of Pd, which were consistent with the peaks of Pd/RGO. And the diffraction peak at 43.3° belongs to the (111) planes of the Cu, suggesting the phase separation between monometallic Pd and Cu in Pd-Cu_(P)_/RGO. The peak positions of the Pd-Cu_(F)_/RGO shifted in comparison with Pd/RGO, suggesting the formation of Pd-Cu alloys [12]. The control experiment of Cu_(F)_/RGO without Pd loading shows additional peaks at 29.6°, 42.4°, 61.4°, and 74.0° corresponding to Cu_2 + 1_O (Cu_2_O with metal excess defects), which confirmed that the copper was loaded on the RGO and oxidized. Moreover, the broad peak at around 25° is detected in each line, which is attributed to the (002) planes of RGO, suggesting the removal of oxygen-containing functional groups from the GO [23].

**Fig. 4:**
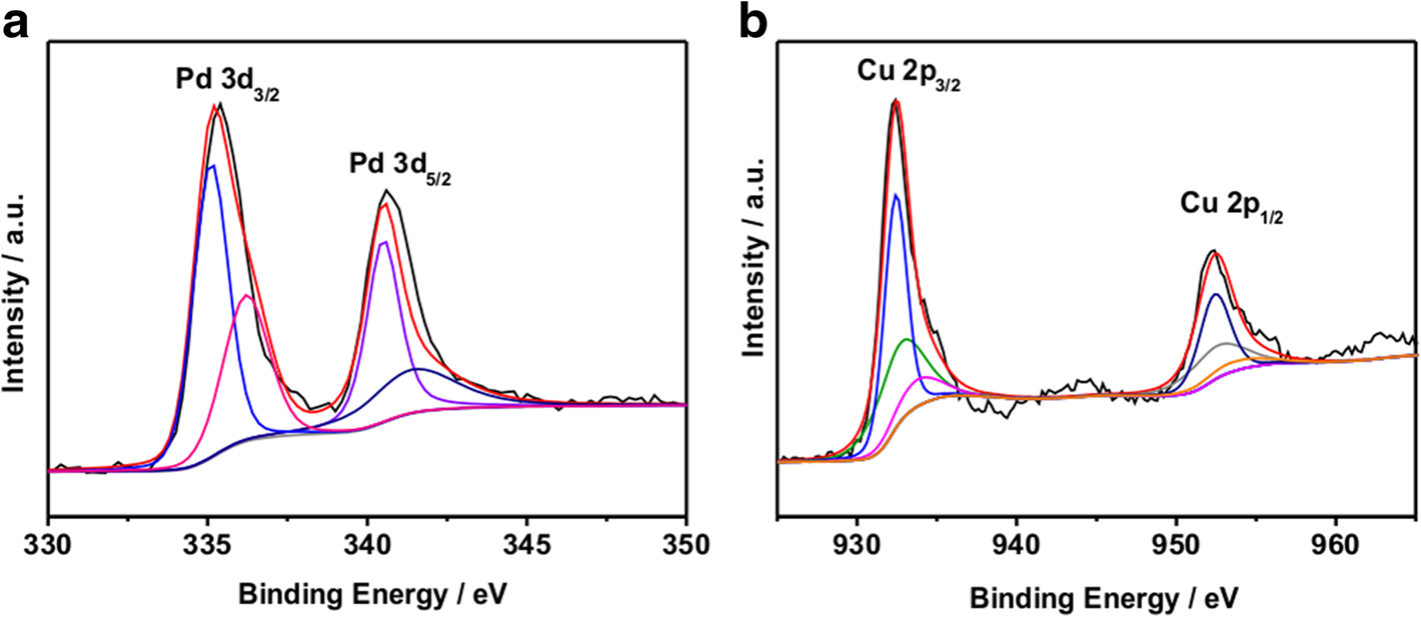
Survey and high-resolution XPS spectra of Pd 3*d* (**a**) and Cu 2*p* (**b**) of Pd-Cu_(F)_/RGO

To further determine the structure, XPS analysis was performed to analyze the surface chemical states and the components of the sample. The high-resolution XPS spectra of Pd 3*d*, Cu 2*p* regions of Pd-Cu_(F)_/RGO are shown in Fig. 4a, b, respectively. The XPS spectrum of Pd was a combination of four peaks that came from Pd at 340.6 and 335.2 eV and PdO at 341.6 and 336.2 eV [4]. In Cu XPS spectrum, the peaks at around 932.6 and 952.6 eV represented Cu 2*p*3/2 and Cu 2*p*1/2, respectively. The Cu 2*p*3/2 and Cu 2*p*1/2 signals were fitted with six peaks that can be due to Cu or Cu_2_O at 932.4 and 952.4 eV, CuO at 933.2 and 953.2 eV, and Cu(OH)_2_ at 934.4 and 955.2 eV, which were partly consistent with the result of XRD.

**Fig. 5:**
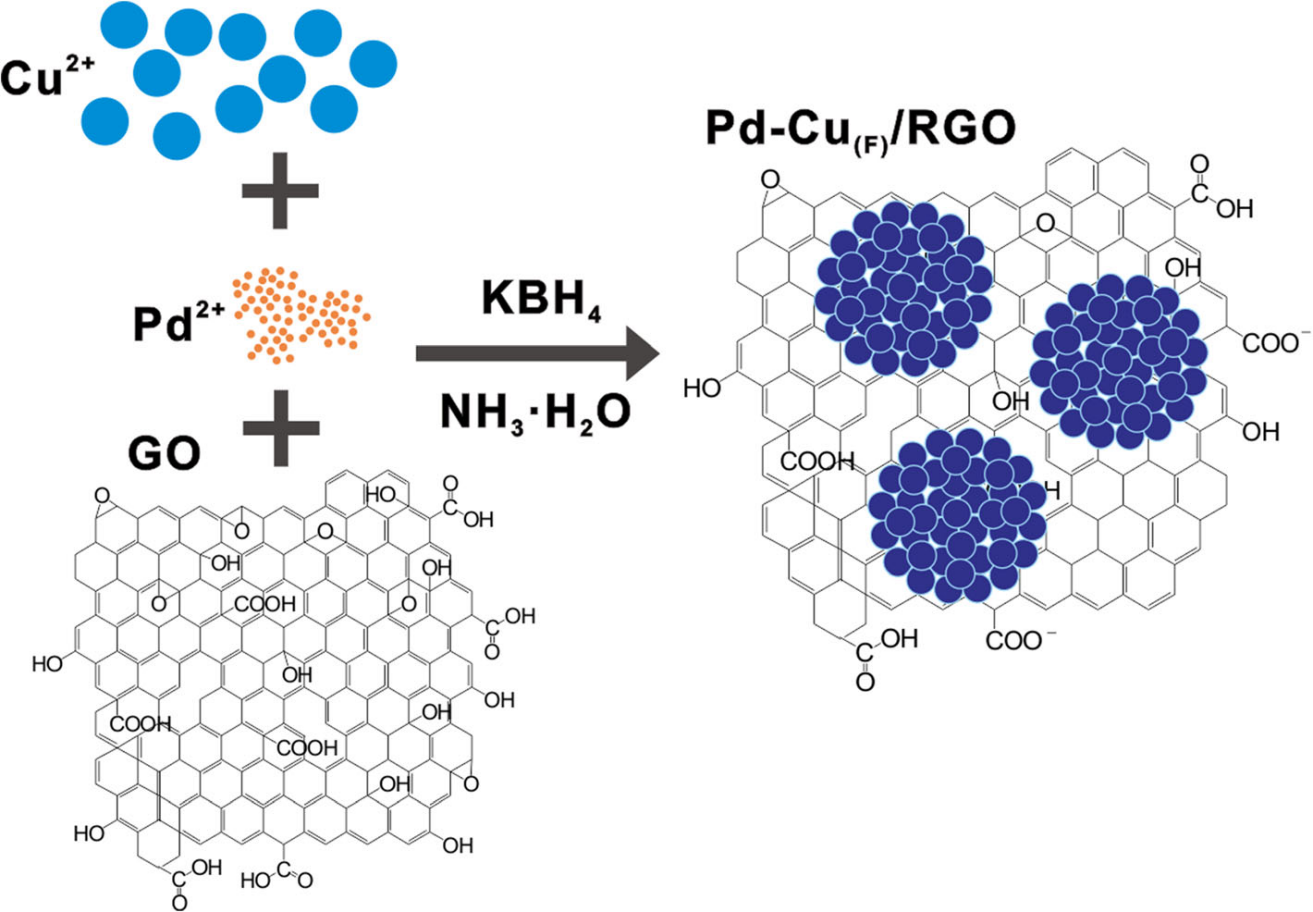
Schematic illustration of the preparation of Pd-Cu_(F)_/RGO nanostructures

According to the XPS data, we proposed the possible formation mechanism as follows: In the solution of ammonia, both Cu^2+^ and Pd^2+^ were coordinated with the ammonia, forming the [Cu(NH_3_)_4_]^2+^ and [Pd(NH_3_)_4_]^2+^, respectively. A fraction of the complexes further combined with OH^−^ to form the metal oxides [24], and the other part, was reduced by KBH_4_ to nanoparticles. During this process, Pd-Cu alloy was formed. It is possible that the adding of ammonia favors the formation of Pd-Cu alloy [25, 26]. We believe the PVP plays a crucial role as a structure-directing agent during the reduction, which is similar to the system of the Pt-Cu under the circumstance of cetyltrimethylammonium bromide (CTAB). CTAB and PVP are usually used to control the nucleation and growth of nanoparticles and influence the reaction rate, resulting in various shapes [27–29]. Meanwhile, GO was reduced to RGO by KBH_4_ and the flower-like Pd-Cu alloy nanoparticles were deposited on the RGO due to the strong interactions between metal or metal oxide nanoparticles and the functional groups of RGO [1]. The schematic of the preparation of Pd-Cu_(F)_/RGO nanostructures was shown in Fig. 5. As for Pd-Cu_(P)_/RGO, according to the works of Zhang QL et al. [1] and Lu L et al. [30], Pd^2^^+^ and Cu^2+^ can be reduced by KBH_4_ and deposited on the RGO, as well as Na_2_CO_3_ by just adjusting the pH of the system.

**Fig. 6:**
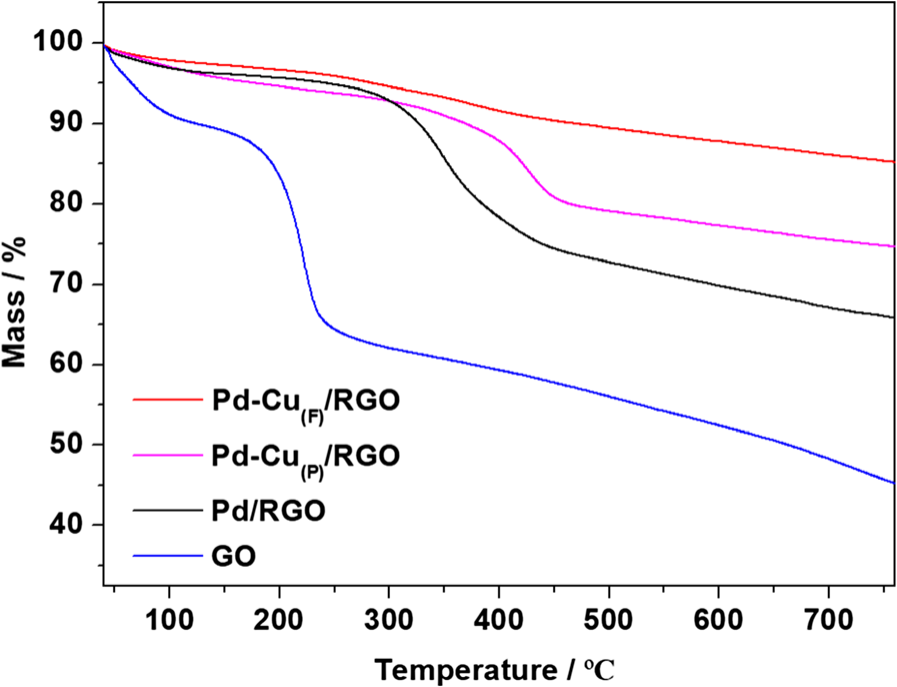
TGA curves of Pd-Cu_(F)_/RGO, Pd-Cu_(P)_/RGO, Pd/RGO, and GO from 40 to 780 °C in air atmosphere

Thermogravimetric analysis (TGA) was performed under a flow of air, and the samples were heated with a heating rate of 10 °C min^− 1^. The analysis was conducted on Pd-Cu_(F)_/RGO, Pd-Cu_(P)_/RGO, Pd/RGO, and GO. The results shown in Fig. 6 illustrated that a weight loss of about 6% of Pd-Cu_(F)_/RGO occurred between 250 and 500 °C, while the weight loss of Pd-Cu_(P)_/RGO was about 14% and Pd/RGO was around 22%. The weight loss of products in air atmosphere at high temperature was probably due to the removal of the remaining oxygen-containing functional groups. The significant weight loss of GO, around 28% between 100 and 300 °C, was mainly due to the removal of oxygen-containing functional groups, just like C–O and C=O. And the weight loss within 100 °C which came from the escape of water molecules between the RGO nanosheets as well as the weight loss above 500 °C was due to the combustion of carbon skeleton [27, 31, 32]. The result indicated the removal of the oxygen-containing functional groups on Pd-Cu_(P)_/RGO and Pd-Cu_(F)_/RGO, which further confirms that GO was efficiently reduced to RGO during the synthesis [23].

**Fig. 7:**
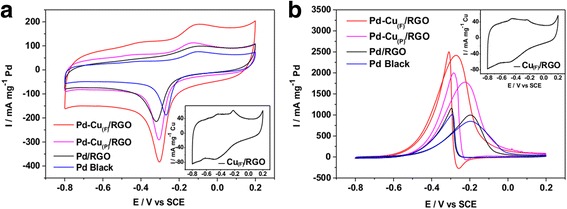
Cyclic voltammograms of the Pd-Cu_(F)_/RGO, Pd-Cu_(P)_/RGO, Pd/RGO, and Pd black. CV plots in 0.5 M NaOH (**a**) and 0.5 M NaOH + 0.5 M C_2_H_5_OH (**b**) at a scan rate of 50 mV s^− 1^. The inserted images are the cyclic voltammogram plots of Cu_(F)_/RGO in 0.5 M NaOH (**a**) and 0.5 M NaOH + 0.5 M C_2_H_5_OH (**b**) at a scan rate of 50 mV s^− 1^

To evaluate the performance of these catalysts for ethanol electrooxidation in alkaline medium, the electrochemical behavior of these catalysts was investigated by cyclic voltammograms (CV) in 0.5 M NaOH solution without and with 0.5 M C_2_H_5_OH. The CVs measured in a N_2_-saturated 0.5 M NaOH solution at a scan rate of 50 mV s^− 1^ were shown in the Fig. 7a. The CV measurements were carried out between − 0.8 and 0.2 V (vs. SCE), and the peaks from − 0.2 to 0 V were contributed by the formation of oxygenated species on the surface of Pd, and the peaks between − 0.4 and − 0.2 V were mainly due to the reduction of PdO, which can release surface sites for ethanol oxidation [2]. The electrochemical active surface area (ECSA) was calculated by the integral area of the reduction of PdO. The ECSA was estimated to be 151.90 m^2^ g^− 1^ Pd for the Pd-Cu_(F)_/RGO, which was larger than that for the Pd-Cu_(P)_/RGO (123.36 m^2^ g^− 1^ Pd), Pd/RGO (102.66 m^2^ g^− 1^ Pd), and Pd black (88.10 m^2^ g^− 1^ Pd).

The CVs of Pd-Cu_(F)_/RGO, Pd-Cu_(P)_/RGO, Pd/RGO, and Pd black in a N_2_-saturated 0.5 M NaOH + 0.5 M C_2_H_5_OH solution were shown in Fig. 7b. The ethanol oxidation current of Pd-Cu_(F)_/RGO (2416.25 mA mg^− 1^ Pd) was higher than that of Pd-Cu_(P)_/RGO (1779.09 mA mg^− 1^ Pd), and much higher than Pd/RGO (997.70 mA mg^− 1^ Pd) and Pd black (847.4 mA mg^− 1^ Pd), which means the Pd-Cu_(F)_/RGO had high activity of ethanol oxidation. There were also different onset potentials of ethanol oxidation among the four catalysts. The onset potential of Pd-Cu_(F)_/RGO was more negative than that of Pd-Cu_(P)_/RGO while much more negative than that of Pd/RGO and Pd black. This observation implied that ethanol molecules can be more easily oxidized on Pd-Cu_(F)_/RGO. We can conclude that the as-prepared flower-like catalysts had better electrochemical performance than the synthetic spherical particle catalysts.

To reveal the role of Cu in the Pd-Cu_(F)_/RGO, a control experiment of Cu_(F)_/RGO without Pd loading was performed under the same condition. As shown in the inset of Fig. 7a, b, there was no obvious peak of ethanol oxidation in the CV carve of Cu_(F)_/RGO in 0.5 M NaOH + 0.5 M C_2_H_5_OH. This result was consistent with the previous reports [4, 12]. The ignorable electrocatalytic activities towards ethanol oxidation of Cu_(F)_/RGO suggested that the Pd acted as the active sites for the electrocatalytic oxidation towards ethanol electrooxidation, and the formation of Pd-Cu alloy can further improve the electrocatalytical activity [33]. The role of Cu in the Pd-Cu_(F)_/RGO in the electrooxidation reaction can be explained by bifunctional effect [12]. Cu is an electron donor atom, while Pd is an electron acceptor. The d-band center shifts when alloying is done between Pd and Cu, and this phenomenon may increase the electrocatalytic oxidation [18, 34, 35]. Therefore, the formation of Pd-Cu alloy will be in favor of ethanol electrooxidation. It is also interesting that the change of morphology from particle to hierarchical flower-like structure also further improves the electroactivity, which is mainly due to the large surface area and increase in the number of catalytic active sites [36].

**Fig. 8:**
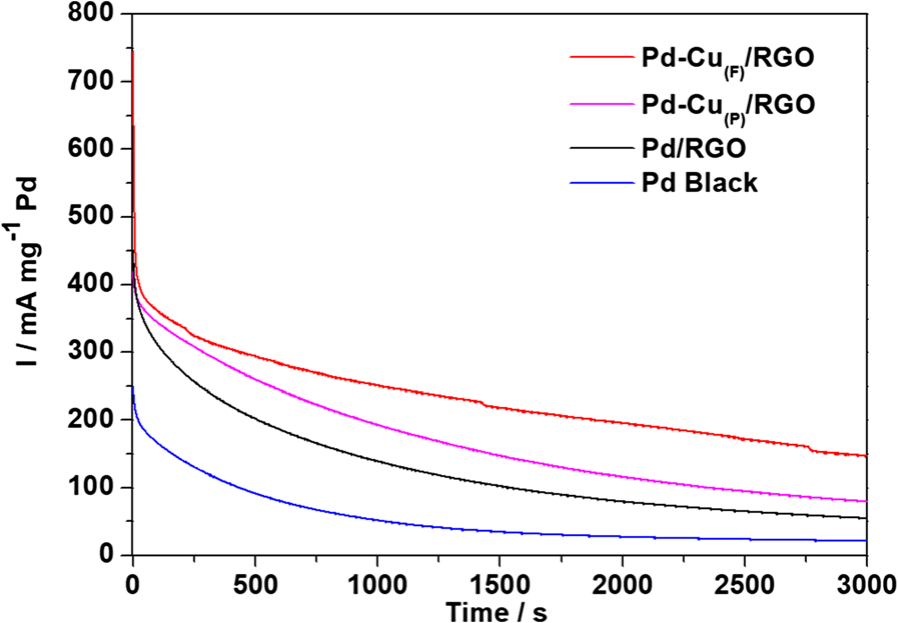
*I*-*T* curves of the Pd-Cu_(F)_/RGO (curve a), Pd-Cu_(P)_/RGO (curve b), Pd/RGO (curve c), and Pd black (curve d) in 0.5 M NaOH + 0.5 M C_2_H_5_OH up to 3000 s at 25 °C

The durability test of these four catalysts were measured in a N_2_-saturated 0.5 M NaOH + 0.5 M C_2_H_5_OH solution for 3000 s at a potential of − 0.35 V, as shown in Fig. 8. Because of the formation of the intermediate species, the initial currents dropped quickly at the beginning [1], and the rate of decay for Pd-Cu_(F)_/RGO was significantly smaller than that of Pd-Cu_(P)_/RGO. The final current after 3000 s of Pd-Cu_(F)_/RGO was much higher than that of Pd-Cu_(P)_/RGO, Pd/RGO, and Pd black under the same conditions, and the values of current density were list in Table 1. These results illustrated the highest long-term electrocatalytic activity of Pd-Cu_(F)_/RGO among the investigated catalysts, which suggested that the formation of hierarchical flower-like morphology and alloying significantly improve the stability of catalysts towards ethanol electrooxidation.

**Table 1:** Peak current densities of various catalysts in 0.5 M NaOH + 0.5 M C_2_H_5_OH at a sweep rate of 50 mV s^− 1^, 25 °C

Catalyst	*I*/mA mg^− 1^ Pd				Final current/initial current
	10 s	1000 s	2000 s	3000 s	
Pd-Cu_(F)_/RGO	441.25	250.75	195.63	146.94	0.33
Pd-Cu_(P)_/RGO	387.52	192.67	115.74	79.10	0.20
Pd/RGO	393.54	138.62	79.00	54.13	0.14
Pd black	213.93	51.03	26.76	20.68	0.10

## Conclusions

In summary, we developed a one-pot synthesis approach for the preparation of a novel hierarchical flower-like Pd-Cu alloy nanocatalysts supported on chemical converted graphene. It is found that the addition of ammonia solution during the preparation of nanocatalysts offers the opportunity to tune the morphology of nanocatalysts and influence the formation of alloy, both of which lead to highly enhanced electrocatalytic activity towards the ethanol oxidation in alkaline medium and better long-term stability of the hierarchical flower-like structure of Pd-Cu_(F)_/RGO than that of the Pd-Cu_(P)_/RGO, Pd/RGO, and Pd black catalysts. The significantly enhanced electrocatalytic activity and durability benefiting from the hierarchical flower-like morphology and Pd-Cu alloy suggest that the Pd-Cu_(F)_/RGO could be promising an electrocatalyst towards ethanol oxidation in DEFCs, revealing the great potential of the structure design of the supporting materials for the future fabrication of nanocatalysts.

## Abbreviations

Cu: Copper
CVs: Cyclic voltammograms
DEFCs: Direct ethanol fuel cells
ECSA: Electrochemical active surface area
GCE: Glassy carbon electrode
GO: Graphene oxide
ICP-OES: Inductively coupled plasma optical emission spectroscopy
Pd: Palladium
RGO: Reduced graphene oxide
SCE: Saturated calomel electrode
SEM: Scanning electron microscopy
TEM: Transmission electron microscope
TGA: Thermogravimetric analysis
XPS: X-ray photoelectron spectrometer
XRD: X-ray diffraction
